# The effect of silver nanoparticles on biological and corrosion behavior of electrophoretically deposited hydroxyapatite film on Ti6Al4V

**DOI:** 10.1007/s10856-024-06784-0

**Published:** 2024-03-25

**Authors:** Hassan Balaei, H. M. Ghasemi, Rouhollah Mehdinavaz Aghdam, B. Cheraghali, Mahmoud Heydarzadeh Sohi

**Affiliations:** 1 School of Metallurgy and Materials Engineering, College of Engineering, University of Tehran, Tehran, Iran; 2 Department of Materials Engineering, South Tehran Branch, Islamic Azad University, Tehran, Iran

## Abstract

Surface modification of titanium and its alloys has been seriously considered by researchers to improve their biological behaviors, in the past few decades. In present research, hydroxyapatite (HA) based composite coatings with different concentrations of 0, 2, 4, and 6 wt% of silver (Ag) nanoparticles were electrophoretically deposited (EPD) on anodized and non-anodized Ti6Al4V, using a direct current at a voltage of 30 V for 10 min at room temperature. The specimens were then characterized by means of X-ray diffraction (XRD) analysis, Fourier transform infrared spectroscopy (FT-IR), scanning electron microscopy (SEM) equipped with energy dispersive spectroscopy (EDS). The cell adhesion images and cell viability results showed that HA-Ag composite coatings significantly promoted the biocompatibility of samples compared with the non-anodized and anodized Ti6Al4V. The viabilities of Mg-63 cells on HA-4%Ag coating and bi-layer coating (HA-4%Ag on anodized specimen) were approximately 91% and they were considered as the best coatings in term of biocompatibility. On the other hand, the antibacterial assessments demonstrated that HA-6%Ag coating had the best antibacterial performance compared with other samples. Furthermore, Tafel polarization curves indicated that corrosion resistance of the bi-layer coating was higher than those of the other specimens. The polarization resistance of this coating was about 7 times more than that of theTi6Al4V alloy.

**Graphical Abstract:**

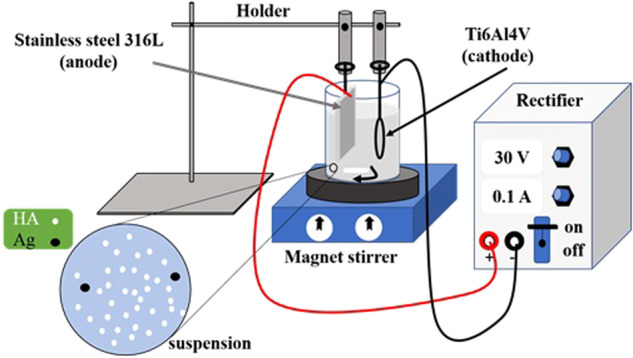

## Introduction

Titanium and its alloys have received great deal of attentions as body implants due to their excellent properties such as high corrosion resistance and suitable biocompatibility [1–3], high strength-to-weight ratio and high fracture toughness [4], the proximity of their elastic module to the body’s natural bone tissue [5] and the presence of passive protective layer on their surfaces [6–8]. Nevertheless, these materials suffer from lack of antimicrobial and low osteogenic properties, which lead to delayed osseointegration as well as complex bacterial infections that may cause rejection and failure of the implant [9, 10]. To overcome these deficiency, various surface modification techniques such as anodizing and deposition of bioactive materials on titanium and its alloys have been studied and developed [11–13]. A number of researchers have focused on the deposition of Calcium-Phosphate (Ca-P) compounds [14–16]. Ca-P bio-ceramics specially HA have widely considered in dental implants, jaw surgery and drug delivery systems [9, 17, 18]. Researches show that around 40% of the body’s natural bone tissue contains apatite minerals [19, 20]. Therefore, it is postulated that by depositing HA on titanium-based implant, the osteogenesis ability of the material, cell attachment to their surfaces as well as the interaction between the bone tissue and the implant material are increased [21, 22]. Surface modification of titanium alloys with HA, apart from promoting the biological behavior of the implants in the body environment has a significant role in controlling of the corrosion reactions [23].

EPD is an appealing technique for the deposition of hydroxyapatite compounds and composite coatings. This process is a cost-effective method in which the thickness and morphology of the coating could be controlled by simply adjusting the voltage and deposition time [24, 25].

Silver nanoparticles are among the materials that are used with HA on the surface of metal implants to reduce bacterial activity [26]. Silver interacts with bacterial proteins and enzymes, which are causing structural damage in the cell walls and bacterial membranes. Silver binds to the DNA and RNA of a bacteria to limit their reproduction. Therefore, HA-based composite coatings containing silver particles have great potential to be applied in bone substitutes or being deposited on metal implants. These composite coatings improve the osteogenesis property at the surface of implant, due to the presence of Ca-P compounds. On the other hand, the presence of silver in the coating, control the activity of bacteria on the implant surfaces [27]. X. Liu et al. [26] showed in their research that the deposition of silver containing Ca-P coating on the surface of metal implants significantly reduces the activity of bacteria and the antibacterial behavior of the composite improved as the concentration of silver increased.

Despite the aforementioned advantages, hydroxyapatite coatings have poor adhesion to the substrate due to their brittle nature [28]. One solution to enhance the adhesion between the coating and the substrate is the presence of an intermediate film such as anodized layer. The surfaces of anodized titanium are rougher and more porous as compared with surfaces of the bare titanium [29]. Therefore, the adhesion of the composite coating to the anodized substrate is more likely to be higher than that of the non-anodized titanium. In addition, anodizing of titanium enhances its corrosion resistance and improve its biocompatibility [30, 31].

Thus, the aim of this research is the investigation of the biocompatibility and corrosion resistance of the hydroxyapatite composite coatings with different amount of silver nanoparticles. These coatings are applied on anodized and non-anodized Ti6Al4V alloy by electrophoretic deposition method and are comparatively studied by evaluating their cell viability, bioactivity, and antibacterial properties.

## Experimental procedure

### Specimen preparation and anodizing process

The specimens used in this study were Ti6Al4V disks with a diameter of 30 mm and thickness of 2 mm with the chemical composition given in Table 1. At first, the surfaces of the specimens were mechanically polished and then ultrasonically cleaned in acetone, ethanol, and deionized water [32]. The anodizing of Ti6Al4V disks was conducted in 1 M sulfuric acid (H_2_SO_4_, Merck, Germany) solution while stirred during the anodizing. The surface areas of the anode (Ti6Al4V) and the cathode (316 L stainless-steel container) were 16 and 50 cm^2^, and their distance was about 5 cm. Samples were anodized using direct current (DC) under potentiostatic conditions at a voltage of 90 V for 30 min at 15 ± 1 °C (using a rectifier device, made in Iran). To keep the temperature of the solution constant, a heat exchanger was used to circulate cooling water around the stainless-steel container during anodizing. After anodizing, the specimens were washed with deionized water and dried at room temperature. Finally, the specimens were heated at 500 °C in a furnace for 1 h to crystallize the amorphous TiO_2_ in anodized layer.

**Table 1:** Chemical composition of Ti6Al4V

Element	Ti	Al	V	Fe	Ni	Cr	Si	C	Zr	Cu	W	Nb	Mo
Wt%	balance	6.28	4.33	0.13	0.01	0.01	0.01	0.01	0.02	0.01	<0.005	<0.005	<0.005

### Suspension preparation and EPD of HA-Ag

**Fig. 1:**
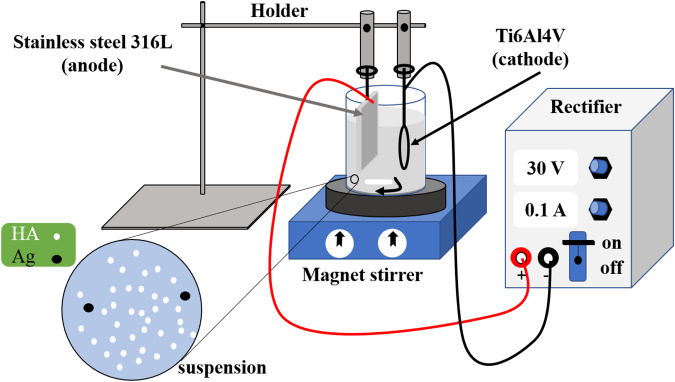
Schematic of EPD process

For preparing the suspension, HA (Ca_10_(PO_4_)_6_(OH)_2_, and silver (Ag) particles (Merck, Germany) with average sizes of 45 and 50 nm, were added to isopropyl alcohol (2-propanol). The suspension was stirred magnetically for 2 h and ultrasonically mixed for 30 min. Subsequently, the pH of the suspension was reduced to 5 by adding nitric acid. The quantities, percentages, and codes of HA and Ag nanoparticles in suspension are represented in Table 2. After preparing the suspension, Ti6Al4V, and anodized samples were used as the cathode, and a 316 L stainless-steel blade was employed as the anode. The distance between the cathode and the anode was about 2 cm during EPD. The schematic of the EPD is shown in Fig. 1. This process was performed using direct current (DC) at a constant voltage of 30 V for 10 min at room temperature (ADAK-PS-303 rectifier, Iran). Finally, the coated specimens were heat treated in a tubular furnace in an argon gas atmosphere at 800 °C for 2 h. The heating rate of the furnace was 4 °C/min.

**Table 2:** The quantities, percentages and codes of HA and Ag nanoparticles in suspension

Substrate	Quantities of particles in suspension (g/l)		Percentages of particles in suspension (%)		Code
	Nano HA	Nano Ag	Nano HA	Nano Ag	
Ti6Al4V	5	0	100	0	HA-0%Ag
Ti6Al4V	4.9	0.1	98	2	HA-2%Ag
Ti6Al4V	4.8	0.2	96	4	HA-4%Ag
Ti6Al4V	4.7	0.3	94	6	HA-6%Ag
Anodized Ti6Al4V	4.8	0.2	96	4	Bi-layer coating

### Characterization of the specimens

The morphological and elemental analyses of the specimens were investigated by a scanning electron microscope (SEM) (QUANTA 200, America) equipped with energy dispersive spectroscopy (EDS) (Silicon Drift 2017, America). The specimens were initially sputter-coated with thin gold films before being characterized by SEM, to make them conductive. The phase structures of specimens were evaluated by utilizing X-ray diffraction (XRD) technique (Rigaku-ultimate IV, America) and using a Cu-Kα (λ = 1.54 A, 40 KV, 30 mA), a scan rate of 4°/min, in the range of 20° < 2θ < 80°, and a step size of 0.04°. The achieved XRD patterns were analyzed by X’Pert High score software. Fourier transform infrared spectroscopy (FT-IR) was also performed (using Bruker-Vector 22, America) in transmittance mode at a range of 400–4000 cm^−1^ to study the functional groups present in the coatings.

### Wettability tests

Wettability tests were conducted to evaluate the hydrophilicity, contact angle, and surface energy of the specimens. A microliter drop was placed on each specimen and photographed by the camera after 10 s. The contact angle of the droplet was determined using Image j software, and the surface energy of the samples was calculated via Eq. 1 [5].

1 $$\tau =\gamma \,\cos (\theta )$$

where θ is the contact angle and γ (surface energy between water and air) is 72.8 mJ/m^2^.

### In vitro tests

#### Cytotoxicity assay

Direct contact method was used to investigate the cytotoxicity of the specimens. At first 5 × 10^3^ MG-63 cells were poured on each sample and incubated at 37 °C for 4 h. After ensuring the adhesion of the cells, 300 microliter of culture medium was added to the surface of the samples. After three days, the medium on the cells was removed as much as possible. 300 microliters of 3-(4,5-dimethylthiazol-2-yl)-2,5-diphenyltetrazolium bromide (MTT) with a concentration of 0.5 mg/ml was poured on each sample. The specimens were then incubated for 4 h at 37 °C with a controlled 5% CO_2_ humidified atmosphere. After removing the solution from the cells, isopropanol was added to appear purple crystals. Then, the solute concentration in isopropanol was calculated using Eliza reader device (Bio Tek ELx808 model, America)- an at a wavelength of 570 nm. Also, a tissue culture plate (TCP) was used as a control. It should be noted that the cytotoxicity test of each series of samples was repeated three times. In this test, solutions with more cells have a high optical density (OD), and the number of cells is identified by Eq. 2 [33].

2 $${\rm{Cell}}\,{\rm{viability}}\,( \% \,{\rm{control}})=\frac{{\rm{mean}}\,{\rm{OD}}\,{\rm{of}}\,{\rm{sample}}}{{\rm{mean}}\,{\rm{OD}}\,{\rm{of}}\,{\rm{control}}}\times 100$$

#### Cell adhesion

In order to study cell adhesion, 20,000 cells were poured on each sample and incubated for 4–5 h. Then, a certain amount of culture medium was added to the sample. After 72 h, the culture medium was removed from its surface. The samples were then washed with Phosphate Buffer Saline (PBS), and 3.5% glutaraldehyde was used for cell fixation. After 2 h, the fixing material was removed, and the specimens were washed with deionized water and alcohol. Finally, the cell adhesion to the samples was examined by scanning electron microscopy.

#### Bioactivity

To study the bioactivity of non-anodized and anodized Ti6Al4V, and coated specimens, a simulated body fluid (SBF, Aprin Company, Iran) was used. The SBF was prepared according to the Kokobo et al. method [34]. At first, the specimens were immersed in 150 ml SBF for 14 days at 37 ± 1 °C. The volume of SBF was calculated according to Eq. 3 [35].

3 $${V}_{S}=\frac{{S}_{a}}{10}$$

where *V*_*s*_ is the volume of SBF (ml) and *S*_*a*_ is the apparent surface area of the sample (mm^2^). Also, apatite formation on the surfaces of the specimens was observed and investigated by scanning electron microscopy equipped with energy dispersive spectroscopy.

#### Antibacterial assay

Staphylococcus aureus (gram-positive bacteria, ATCC 25923) and Escherichia coli (gram-negative bacteria, ATCC 25922) were used to evaluate the antibacterial properties. The antibacterial properties of the samples were conducted by the Agar disc diffusion method. For this purpose, a suspension of the studied bacteria was first cultured on Müller–Hinton agar plates. The specimens were then placed on the agar surfaces under sterile conditions. 5 mg Ciprofloxacin antibiotic discs were also used as the internal control. Finally, the Müller–Hinton agar plates were incubated for one day at 37 °C. After this period, the diameter of the inhibition zone of the tested specimen was measured and recorded by a particular ruler. Also, the surface area of the inhibition zone was calculated by Eq. 4.

4 $${\rm{Surface}}\,{\rm{area}}\,{\rm{of}}\,{\rm{inhibition}}\,{\rm{zone}}\,=\pi ({\rm{D}}^{2}-{\rm{D}}_{0}^{2})/4$$

In this equation, D is the diameter of the inhibition zone and D_0_ is diameter of samples which is 30 mm for the Ti6Al4V discs and anodized and coated specimens and 8 mm for the control discs.

### Corrosion tests

Electrochemical assessment of non-anodized and anodized Ti6Al4V and the coated specimens with a surface area of 0.785 cm^2^ were performed by the conventional three-electrode electrochemical cell method. Samples, a platinum electrode, and a saturated Ag/AgCl electrode were used as the working, counter, and reference electrodes, respectively [36]. Corrosion tests were carried out in PBS solution as a simulated body fluid (pH = 7.4) at room temperature. PBS solution was made according to the literature [37], with a chemical composition of 8.0 g/L NaCl, 1.15 g/L Na_2_HPO_4_, 0.2 g/L KCl, and 0.2 g/L KH_2_PO_4_ in distilled water. At first, the specimens were immersed in the PBS solution for an h to stabilize open circuit potential (OCP). Polarization tests were then conducted with a scanning rate of 1 mV/s by a 302 N Auto lab potentiostat/galvanostat coupled with NOVA 1.9 software.

## Results and discussion

### Characterization of anodized layer

**Fig. 2:**
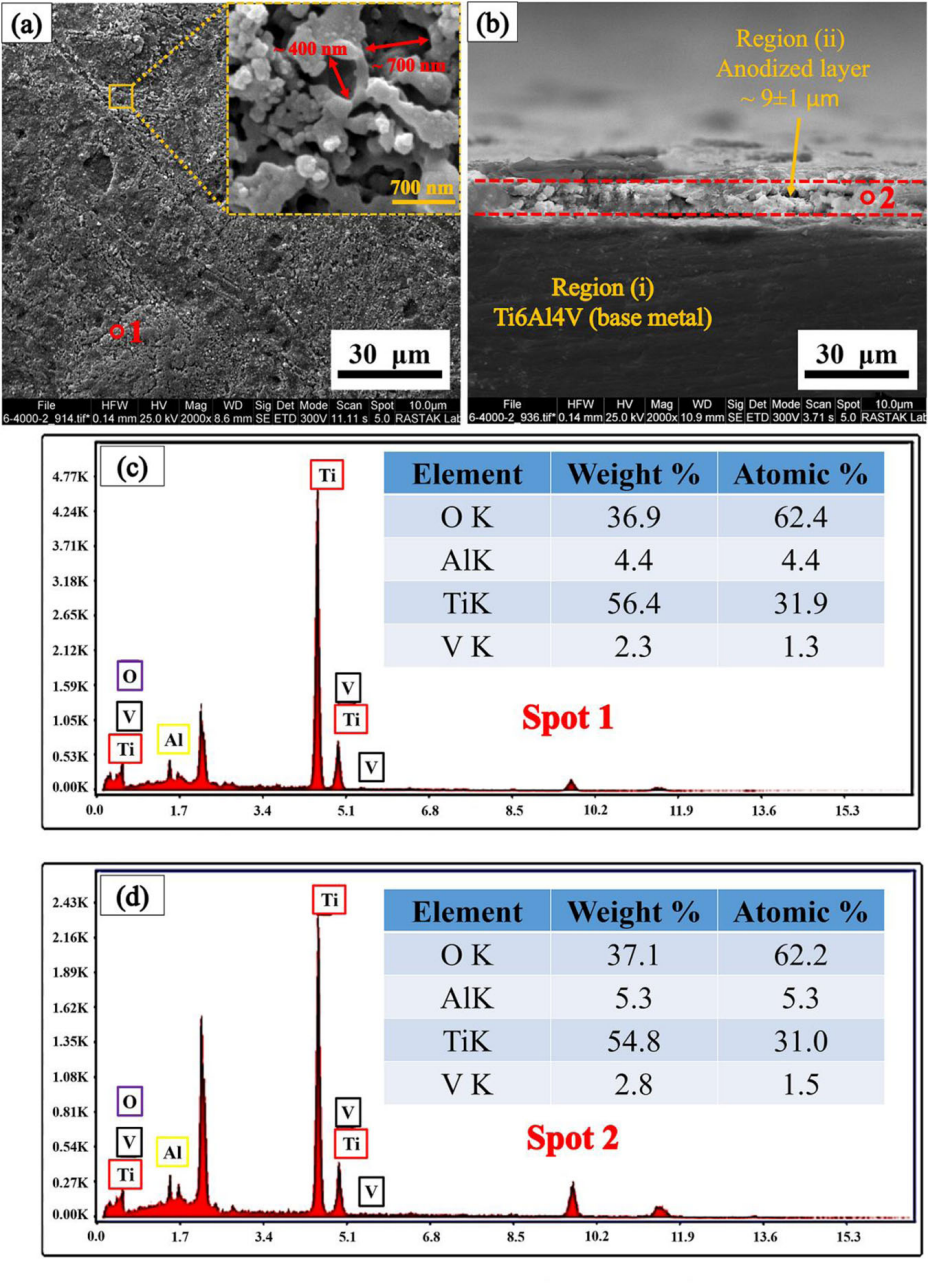
SEM images of surface morphology of anodized Ti6Al4V (**a**), cross-section of anodized layer (**b**), EDS analyzes of surface (**c**) and cross-section (**d**) of anodized layer

The surface and cross-sectional micrographs of the anodized Ti6Al4V together with their respected EDS results are given in Fig. 2a–d, respectively. Figure 2a reveals porosities in the anodized layer. The presence of the pores in this layer can lead to better locking and adhesion of the post composite coatings [38]. During anodizing process, oxygen is formed at the metal-oxide layer interface, and the release of this gas can locally damage the oxide layer and cause porosity [39]. According to Fig. 2a, the diameter of the surface pores in the anodized layer is about 600 ± 100 nm, which can fix biocompatible nanoparticles with dimensions below 100 nm. The cross-sectional image of the anodized Ti6Al4V is presented in Fig. 2b. The figure shows that the thickness of the oxide layer is about 9 ± 1 μm. The elemental analysis and EDS pattern obtained from the surface and cross-section of the anodized layer have also been illustrated in Fig. 2c, d, respectively. Based on these analyses, the atomic percentage of oxygen versus titanium $(\frac{{\rm{atm}} \% {\rm{O}}}{{\rm{atm}} \% {\rm{Ti}}})$, in both cases, was approximately about 2. This ratio confirms the existence of the TiO_2_ oxide layer after the anodizing. Figure 2b indicates the presence of microcracks in the cross-sectional image of the anodized layer, which can be due to the presence of internal stresses in this layer [40]. Another reason for the formation of these microcracks can be attributed to the increase in temperature at the metal/anodized layer interface caused by high voltage during the anodizing process, which leads to the presence of weak points in this layer and makes a larger path for spark discharge [29].

### Characterization of HA-Ag coatings

**Fig. 3:**
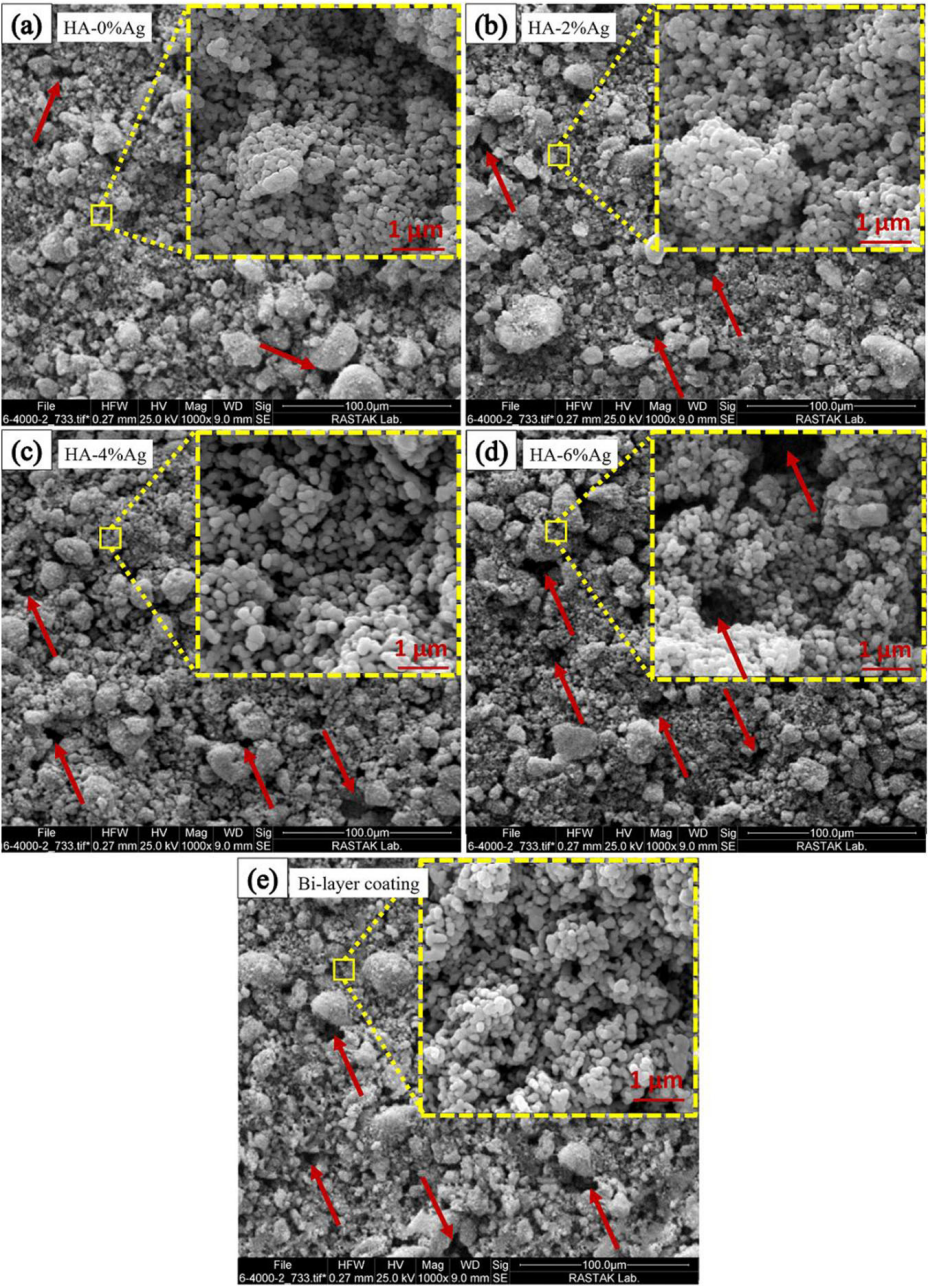
SEM images of Surface morphology of coatings, (**a**): HA-0%Ag, (**b**): HA-2%Ag, (**c**): HA-4%Ag and (**d**): HA-6%Ag on Ti6Al4V and (**e**): HA-4%Ag on anodized Ti6Al4V

SEM images of composite coatings are shown in Fig. 3a–e. These figures show that as the concentration of silver in the EPD suspension increases, the cavities in the surface of the coatings also increase. These cavities are shown by red arrows in Fig. 3. This increase may be due to the agglomeration of the silver nanoparticles in the EPD suspension. These agglomerated silver particles are placed on the surface not in a uniform but in a bunch and irregular form, and the number of cavities on the surface increases.

**Fig. 4:**
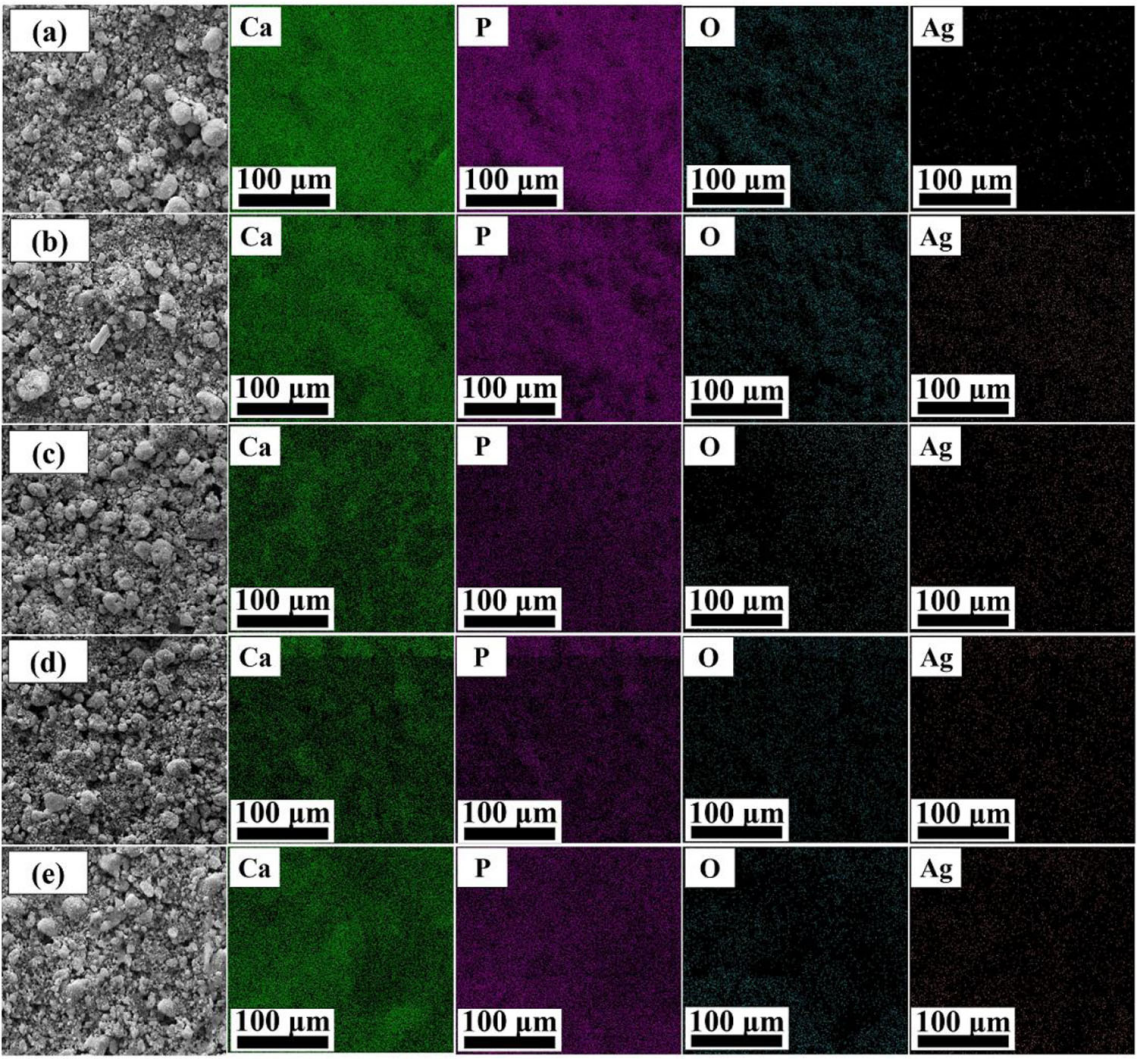
SEM images and their respected EDS mapping of surface of coated samples, (**a**): HA-0%Ag, (**b**): HA-2%Ag, (**c**): HA-4%Ag and (**d**): HA-6%Ag on Ti6Al4V and (**e**): HA-4%Ag on anodized Ti6Al4V

**Fig. 5:**
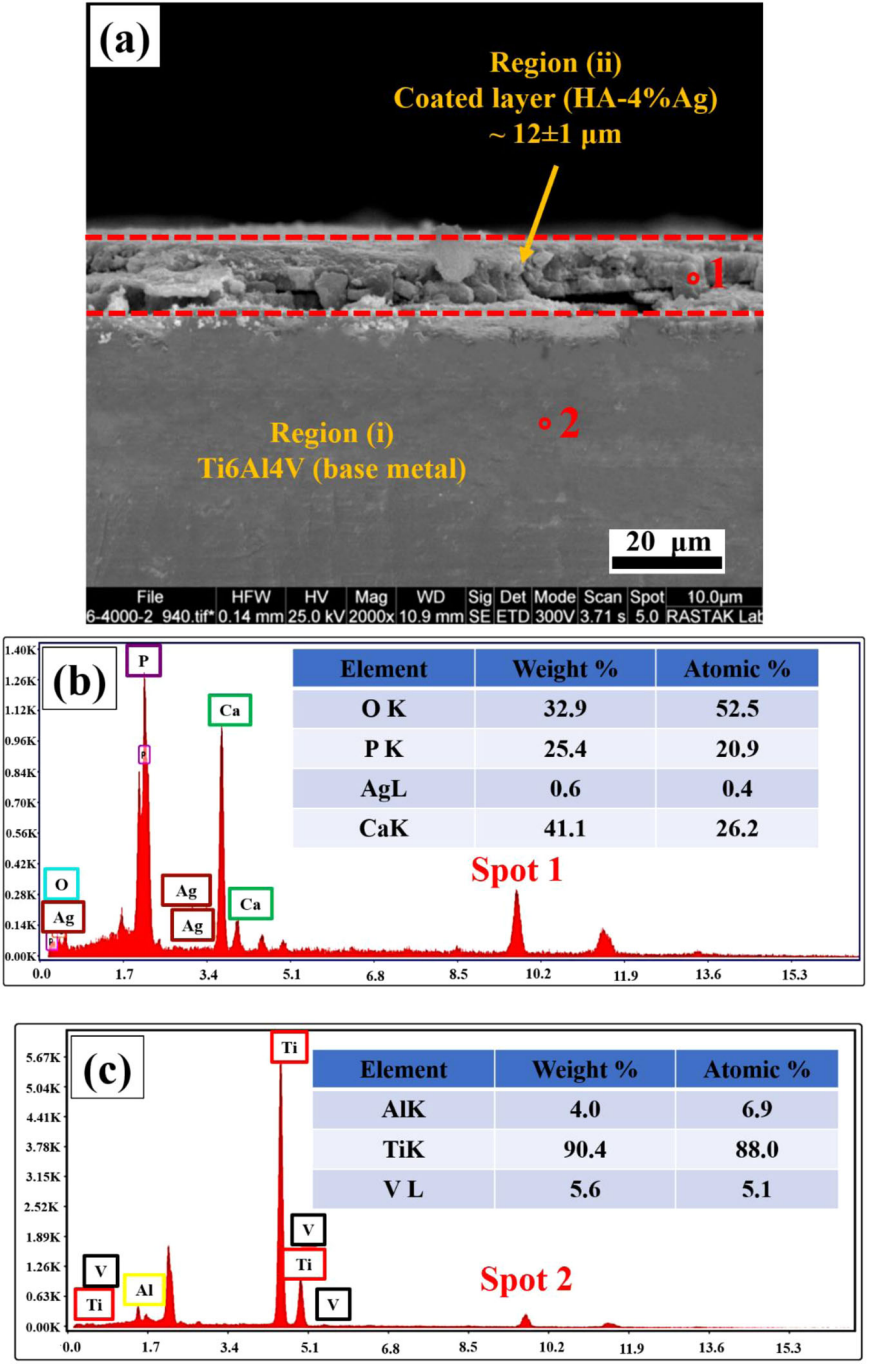
**a** SEM image of cross-section of HA-4%Ag composite coating on Ti6Al4V, (**b**): EDS results of spots 1 in Fig. 5a, (**c**): EDS results of spots 2 in Fig. 5a

**Fig. 6:**
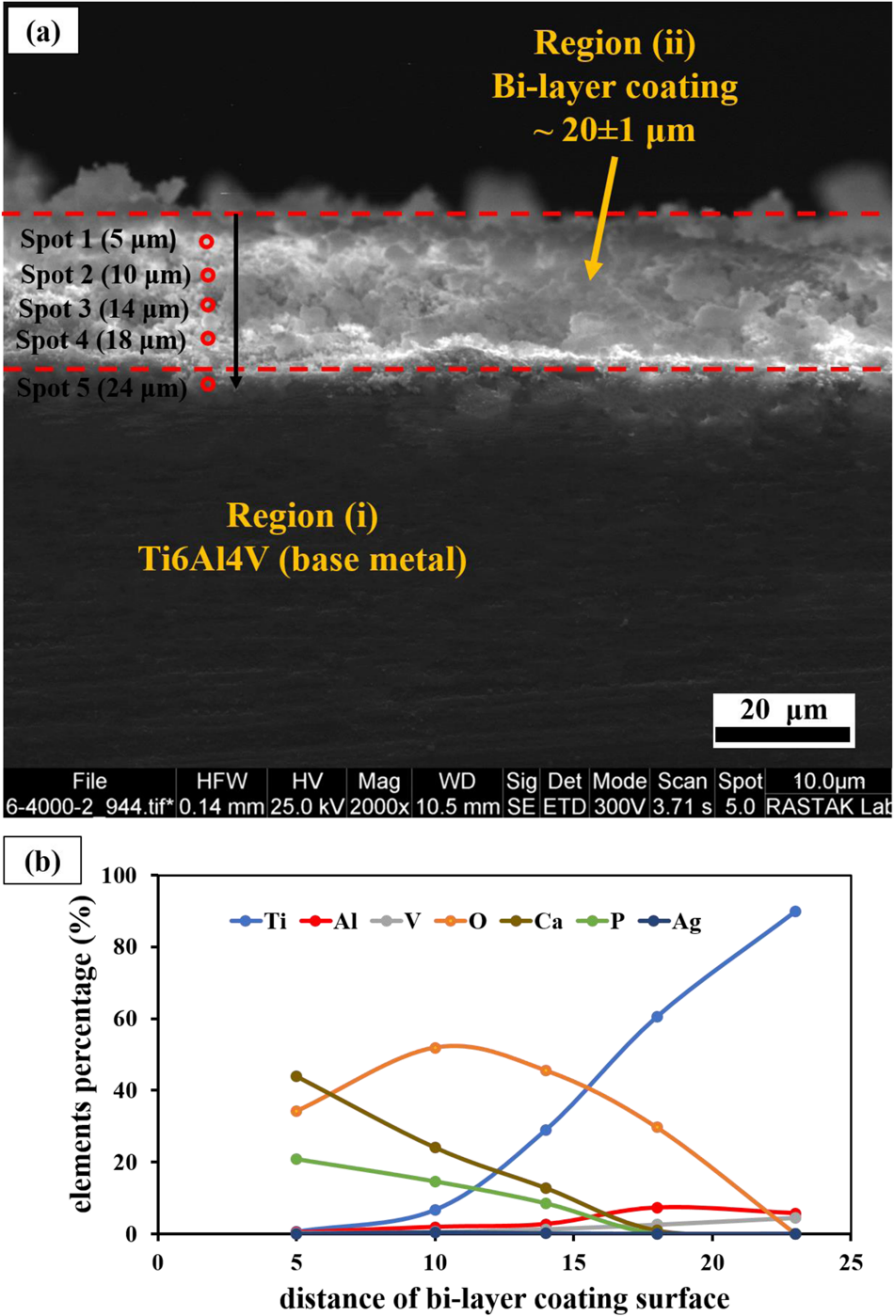
**a** SEM image from the cross-section of bi-layer coating and (**b**) Variation of chemical composition from the bi-layer surface into the depth

The EDS mappings of the coatings are also illustrated in Fig. 4a–e. These maps indicate the distribution and presence of calcium, phosphorus, oxygen (related to hydroxyapatite nanoparticles), and silver compounds in the coatings. The SEM Image of cross-section of the HA-4%Ag composite coating is shown in Fig. 5a. As the figure shows, the thickness of the layer is about 12 ± 1 μm. Figure 5b, c displays the EDS results of the coated layer and the titanium substrate, respectively. Figure 6a also demonstrates the cross-section of the bi-layer (anodized/HA-4%Ag) coating. The thickness of this coating is about 20 ± 1 μm. This figure does not show a sharp interface between the anodized layer and the composite coating. This observation indicates that the HA and Ag nanoparticles as the coating components have diffused into the cavities of the anodized layer (Fig. 2a). The variation of chemical composition from the bi-layer surface into the depth is also illustrated in Fig. 6b. The high quantity of oxygen at distances of 10 and 14 µm from the surface, can be attributed to the simultaneous presence of hydroxyapatite particles and anodized layer, both of which contain oxygen. Also, oxygen in distances of 5 and 18 µm is only related to HA and anodized layer, respectively.

**Fig. 7:**
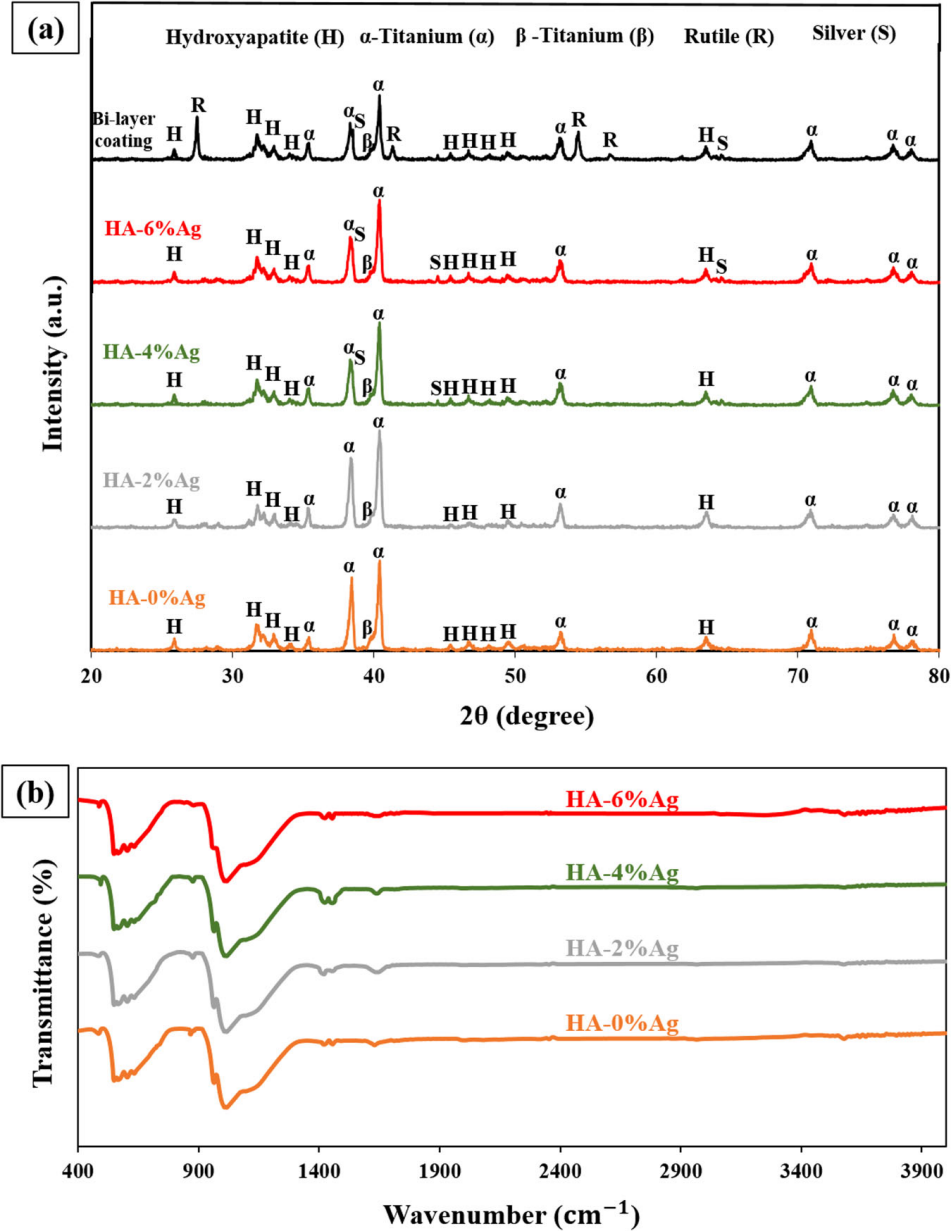
**a** XRD patterns of all samples, (**b**) FT-IR spectra of HA-Ag composite coatings

XRD patterns and FT-IR results of the coatings are illustrated in Fig. 7a, b. Based on the pattern in Fig. 7a, all samples have typical HA diffraction peaks. These peaks are according to the standard JCPDS database of HA (PDF No:09-0432). The diffraction peaks of Ag nanoparticles were observed in the composite coatings with 4 and 6% silver. These diffractions were around 2θ = 38°, 2θ = 44°, and 2θ = 64° for (111), (200), and (220) crystallographic planes, respectively (refer to PDF No: 65-2871). The lack of silver peaks in the HA-2%Ag coating is due to the low concentration of the Ag particles.

FT-IR spectra of HA-Ag composite coatings are displayed in Fig. 7b. As observed in the figure, the peaks around 1090, 1036, and 962 cm^−1^ are related to stretching modes. Also, the presence of the peaks in 602, 565, and 473 cm^−1^ are attributed to the bending bonds of PO_4_^3−^. The peaks appeared in 3570 and 630 cm^−1^ are attributed to stretching and bending bonds of the hydrogel ions (OH^−^) present in the HA crystals. The peaks at 1453, 1421, and 875 cm^−1^ belong to carbonate groups (CO_3_^2−^) of the appetite lattice. Functional groups of carbonates formed due to the presence of CO_2_ gas during the synthesis of HA nanoparticles [21]. The peak in 1639 cm^−1^ also was assigned to the water absorption by HA. These peaks correspond to characteristic bonds for HA reported in the literature [41].

### Investigation of biocompatibility

#### Cell viability evaluation

**Fig. 8:**
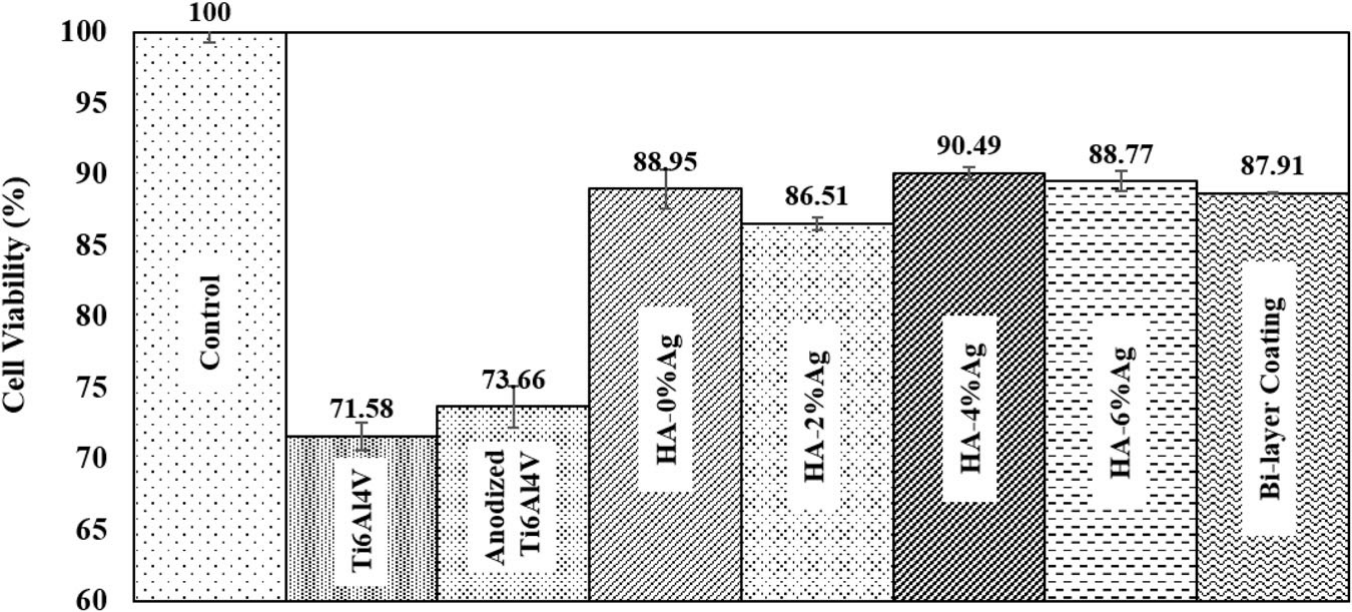
MTT results and the cell viabilities of samples after 72 h’ cell culturing. The results were normalized as compared to the control data, which was considered to have 100% cell viability

MTT assay was used to evaluate the viability of MG-63 cells after 72 h of cell culture. By increasing the cell viability on a surface, the biocompatibility of that surface also increases [42]. The results are presented in Fig. 8. According to the figure, the biocompatibility of all samples was lower than the control specimen. In addition, the biocompatibility of Ti6Al4V sample and the anodized specimen were about 72% and 74%, respectively. This shows that anodizing process had no significant effect on cell viability as compared to Ti6Al4V.

By applying the HA-Ag coatings on Ti6Al4V, the cytotoxicity of the samples decreased, and their biocompatibilities were increased (Fig. 8). Different parameters, such as the release rate of the existing elements and the degree of hydrophilicity are influential on the biocompatibility [43]. The increase in cell viability in the coated samples may be attributed to the presence of the biocompatible HA coatings on their surfaces. Because the cultured cells can quickly proliferate on these biocompatible materials. Also, the presence of ceramic-based material (HA) on the surface increases the cell viability and biocompatibility by reducing the release of elements in the substrate.

The amount of silver nanoparticles in the coatings (0, 2, 4, and 6%wt in suspension) does not significantly increase or decrease biocompatibility. Some researchers have represented the low effect of silver on cell viability in composite coatings [44–46]. Although the concentration of silver in the composite coatings has small or no effect on cell viability, but HA-4%Ag coating had the highest biocompatibility. The cell viability on this coating was approximately 90%. Finally, the optimized coating (HA-4%Ag) was applied on the anodized Ti6Al4V (Bi-layer coating). Based on the results of the MTT assay, the percentages of the biocompatibility of HA-4%Ag and bi-layer coatings were almost the same. Overall, it appears that the HA coatings with different concentrations of silver can improve the biocompatibility of the samples.

In HA-Ag composite coatings, the Ca lattice site in HA can be replaced by Ag nanoparticles. This causes some changes in the crystal structure of HA and leads to the formation of very low amounts of amorphous solid particles. Nonetheless, Silver nanoparticles have a more effective antibacterial function due to their large surface area and lower solubility than silver salts and create better contact with microorganisms [47]. Also, according to Fig. 8, the cell viability of HA-Ag nanocomposite is more than Ti6Al4V sample. So, it can be concluded that simultaneous role of chemical and physical properties of the hydroxyapatite and silver nanoparticles increase the cell proliferation and biocompatibility. It should be noted that B. Tian et al. [48] showed in their research that silver nanoparticles-doped hydroxyapatite coatings improve the cell viability of Ti6Al4V sample.

#### Cell adhesion assesment

**Fig. 9:**
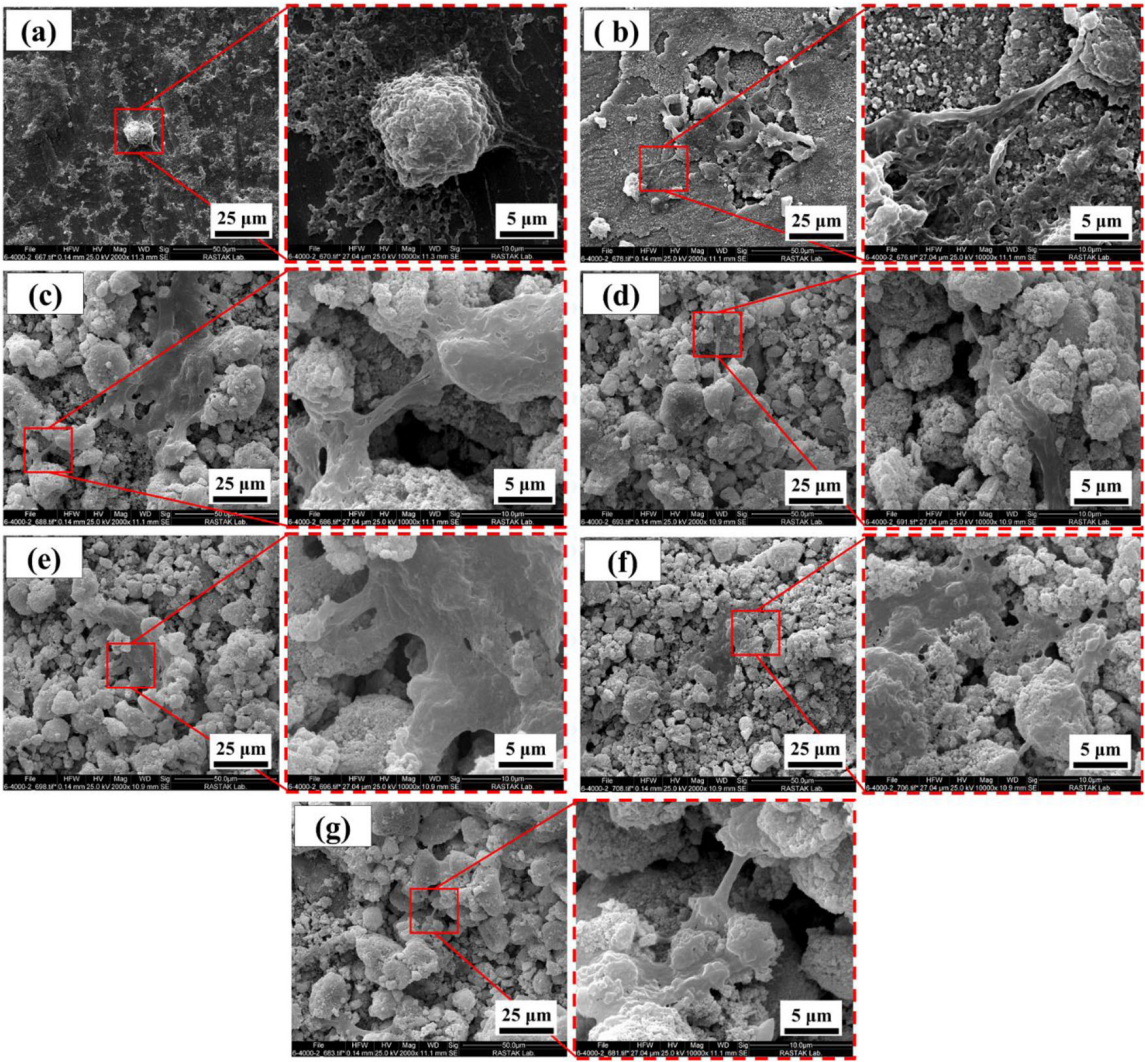
SEM images of MG-63 cells on the surface of samples, (**a**) Ti6Al4V, (**b**) Anodized Ti6Al4V (**c**) HA-0%Ag, (**d**) HA-2%Ag, (**e**) HA-4%Ag, (**f**) HA-6%Ag and (**g**) Bi-layer coating

Cell adhesion to the surface has three steps: (1) attaching the cells to a surface, (2) spreading the cell on the surface, and (3) creating a bond between the cell and the surface [49]. Figure 9a–g illustrates the adhesion of MG-63 cells on the surfaces of different samples. After cell attachment, a good bond is created between the osteoblast cells and the surfaces of the samples (Fig. 9a–g).

It can be deduced from Fig. 9 that the flatting of osteoblast cells on the surface of Ti6Al4V was lower than those of the other samples. This may be attributed to the higher release of metal ions from the surface of Ti6Al4V. Because ion release can reduce the spread of osteoblast cells on the surface. Also, cavities and pores on surfaces facilitate the attachment of cellular philopodia to them [50]. According to Fig. 9, the tentacles of the cells can adhere to the surfaces of anodized and coated specimens, which have higher cavities and holes than those of the Ti6Al4V.

Some researchers have shown that the flatting of cells on substrates depends on surface wettability, surface chemistry, and surface energy [42, 51]. Due to the high wettability of the anodized layer surfaces, the spreading of cells on anodized surfaces is higher than Ti6Al4V. In other words, the spreading of cells on the hydrophobic surfaces is weaker.

Overall, actin polymers and associated proteins constitute the cytoskeleton, which is a highly dynamic structural network. This structure provides variety of essential biological activities including intracellular transport and migration [52]. Figure 9 shows that on surfaces of HA-Ag composite coatings, cells were polygonal and well distributed; however, on Ti-6Al 4V they were a spindle and spherical shape, and showed poor spreading.

**Fig. 10:**
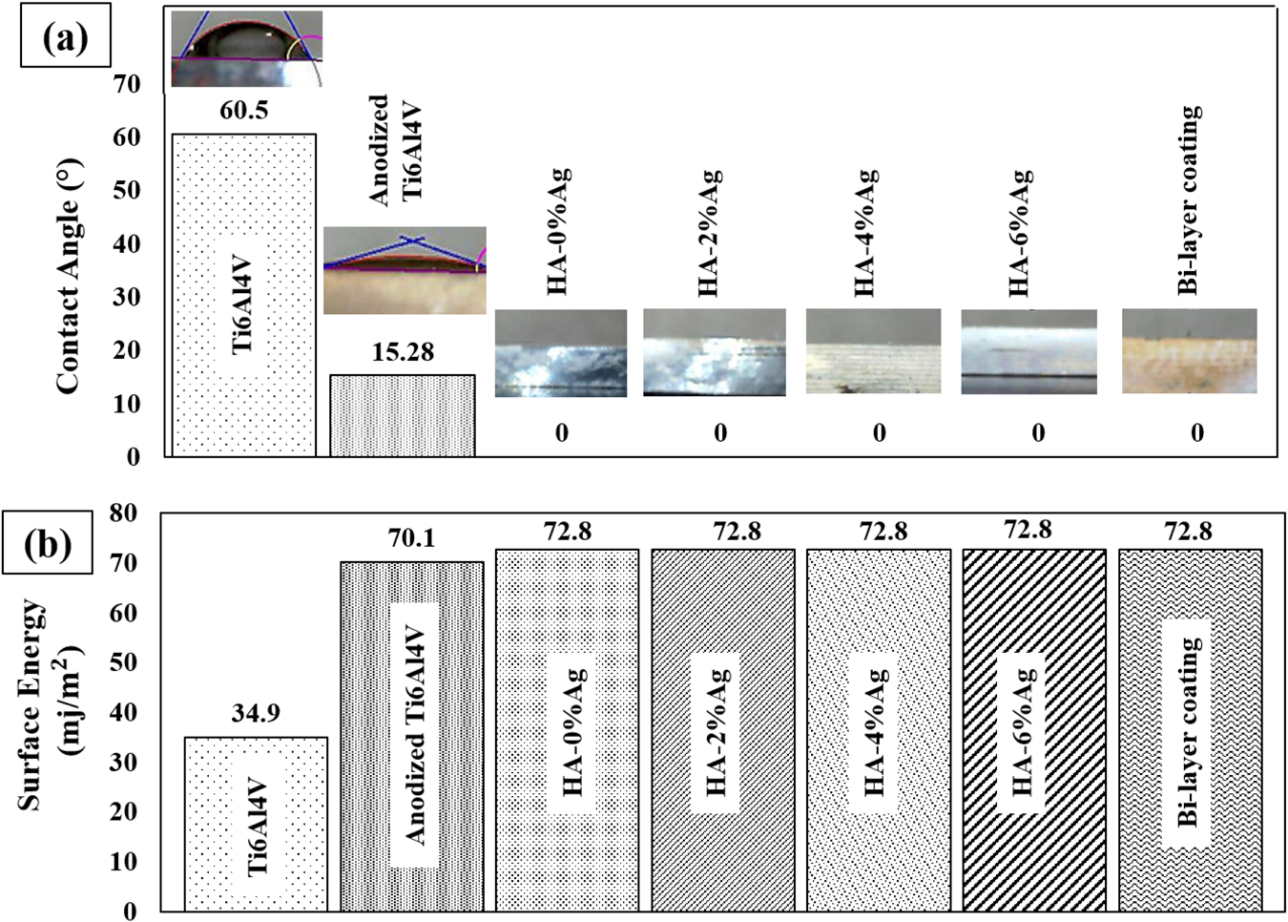
**a** Contact angle and (**b**) Surface energy of samples

According to Eq. 1 and Fig. 10a, b, reduction of the contact angle results in increase in the surface energy. The decrease of contact angle and increase of hydrophilicity and surface energy causes most in vitro activation of cells. Because of the high value of the surface energy and hydrophilicity of the coated samples (Fig. 10b), osteoblast cells can spread on the surfaces of the coatings. So, the cell adhesion images (Fig. 9) of samples confirm the wettability results, shown in Fig. 10a.

Figure 10a shows that the contact angle of non-anodized and anodized Ti6Al4V was approximately 60 and 15°, respectively. The lower value of the contact angle for the anodized layer was related to their surface cavities of that were higher than those of the Ti6Al4V (Fig. 2). Increasing surface cavities and decreasing contact angle are preferred for biocompatibility [53]. By applying HA coatings with different concentrations of Ag nanoparticles, this angle reaches to 0°. The reason for reducing the value of the contact angle in the coated samples is probably attributed to the presence of the hydroxyl groups on their surfaces. In other words, hydroxyl groups increase the hydrophilicity and wettability of the specimens. The presence of cavities and holes on the coated samples can also reduce the contact angle. In the surfaces containing cavities, in equal-size zones, the area is more than the surface without cavities. As a result, water droplets can better spread on a larger surface. This subsequently reduces the contact angle and increases wettability, hydrophilicity, and surface energy.

#### Bioactivity investigation

The bioactivity of the surfaces is determined by their ability to form apatite during immersion in simulated body fluid (SBF). It should be noted that the apatite formed during the immersion test has a porous structure and is spongy or flaky [35]. In other words, the morphology of apatite formed after the immersion test is different from the HA formed by the EPD process, which is shown in Fig. 3. The SEM images of generated apatite from SBF are similar to the images that have presented by T. Kokubo and H. Takadama [35].

**Fig. 11:**
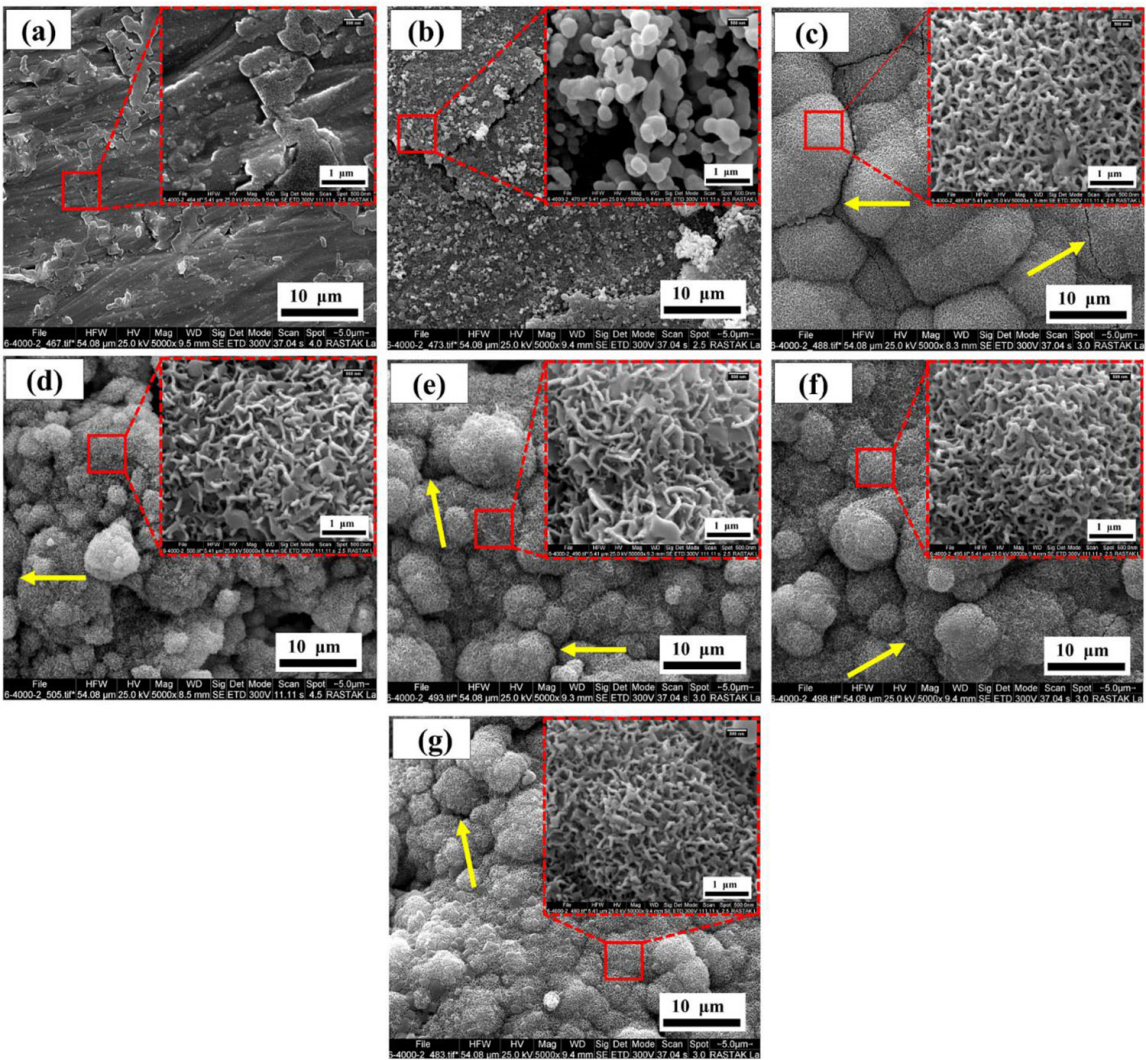
SEM images of surfaces of immersed samples in SBF after 14 days, (**a**) Ti6Al4V, (**b**) Anodized Ti6Al4V (**c**) HA-0%Ag, (**d**) HA-2%Ag, (**e**) HA-4%Ag, (**f**) HA-6%Ag and (**g**) Bi-layer coating

The SEM images of the samples immersed for 14 days in SBF are shown in Fig. 11a–g. According to these images, the surface bioactivities of non-anodized and anodized Ti6Al4V are very weak. This is due to the lack of apatite generation during the immersion period in simulated body fluid. But by applying HA-based composite coated specimens, the bioactivity of the samples increased significantly. After the immersion test, apatite is formed on the coated specimens, which indicates the high bioactivity of the coated surfaces. Therefore, HA-based composite coatings can significantly improve the bioactivity of the material. The high bioactivity of the coated samples compared to those of the non-anodized and anodized Ti6Al4V is probably due to the presence of HA on them. HA is similar to natural bone tissue and increases the osteogenesis of the surface [21].

Study of Fig. 3c–g shows that some micro cracks are seen on the surfaces of the samples after the immersion test. Yellow marks illustrate these zones in Fig. 3. Most likely, these micro cracks are formed during the sintering of the samples after the EPD process on the surfaces of HA-based composite coated specimens. It should be mentioned that after the formation of apatite on these samples during the immersion test, these micro cracks were not disappeared.

#### Antibacterial properties

**Fig. 12:**
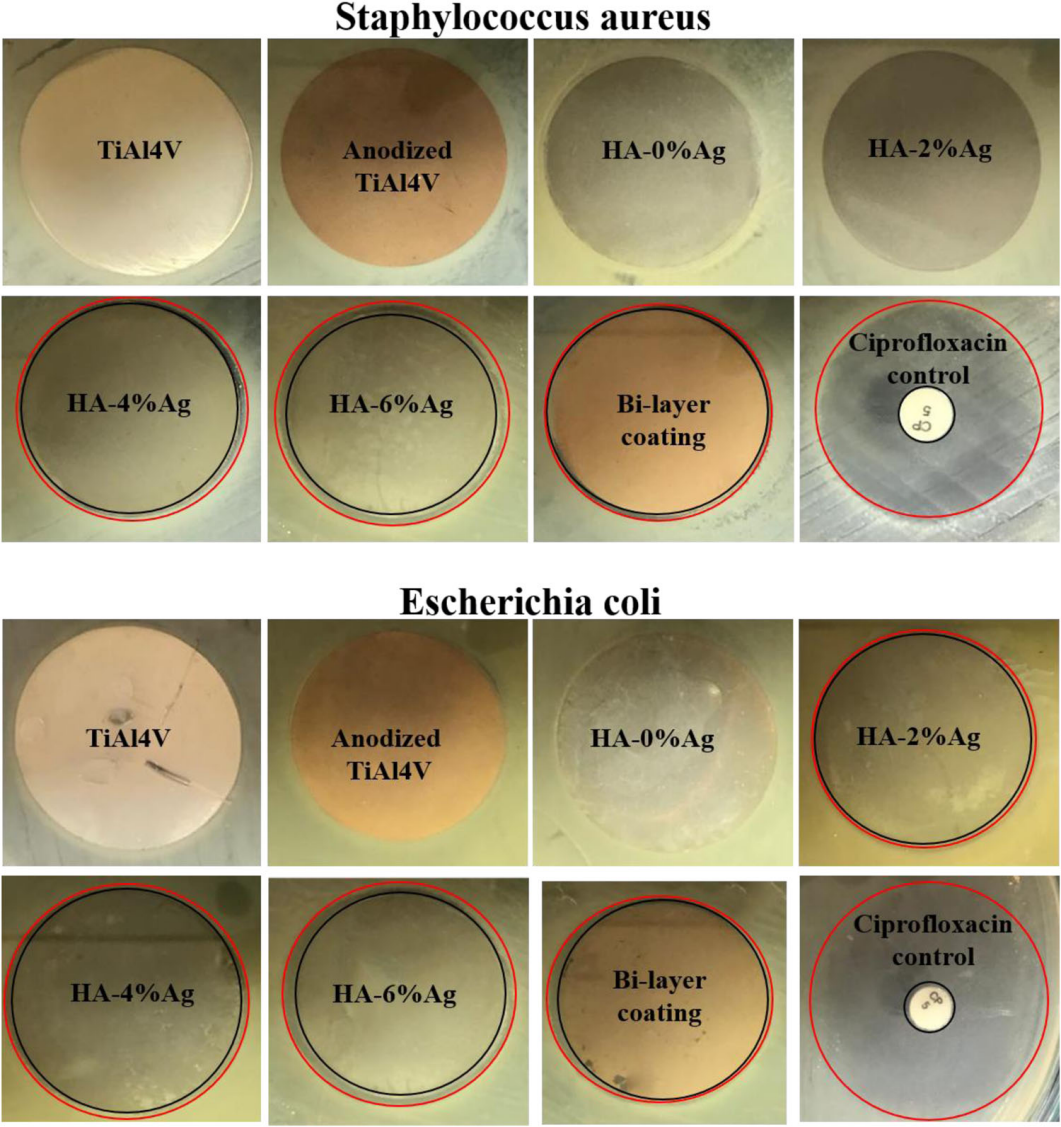
Photographs of inhibition zones of samples and ciprofloxacin control using staphylococcus aureus and Escherichia coli bacteria by disk diffusion assay (diameters of the samples and control specimen were 30 and 6 mm, respectively)

The antibacterial assessments of Ti6Al4V, anodized, and coated samples were conducted by agar disk diffusion method. In these tests, Staphylococcus aureus (gram-positive bacteria) and Escherichia coli (gram-negative bacteria) were used. The results of antibacterial tests are shown in Fig. 12. In this figure, the area between the black and red circles illustrates the inhibition zone. The diameters and surface areas of inhibition zones for the samples and controls are also represented in Table 3.

**Table 3:** The diameter and surface area of inhibition zone of samples in presence of Staphylococcus aureus and Escherichia coli bacteria

Sample	Staphylococcus aureus		Escherichia coli	
	Inhibition zone		Inhibition zone	
	Diameter (mm)	Surface area (mm^2^)	Diameter (mm)	Surface area (mm^2^)
Ti6Al4V	0	0	0	0
Anodized Ti6Al4V	0	0	0	0
HA-0%Ag	0	0	0	0
HA-2%Ag	0	0	31	48
HA-4%Ag	32	97	32	97
HA-6%Ag	33	148	33	148
Bi-layer coating	31	48	32	97
Control	34	857	36	967

According to Fig. 12 and Table 3, it can be seen that all of the specimens have lower antibacterial properties than those of the control samples. Because the diameter and the surface area of the inhibition zone for ciprofloxacin controls were higher than those of the other samples. Also, the diameters and surface areas of the inhibition zone for non-anodized and anodized Ti6Al4V and HA-0%Ag coating were about 0 mm and 0 mm^2^, respectively. These values indicate that these samples lack antibacterial properties.

The reported results in Table 3 shows that increasing of the amount of silver in the coating suspension increases the antibacterial properties. According to this Table, after ciprofloxacin controls, the highest antibacterial properties achieved in HA-6%Ag coating. This is due to the antibacterial nature of silver nanoparticles used in the composite coatings. For example, Z, Geng, et al. [54] demonstrated that the presence of silver particles in calcium-phosphate coating improved its antimicrobial properties.

The diameter and surface area of the inhibition zone of HA-2%Ag composite coating that was calculated by using the Staphylococcus aureus bacteria was about 0 mm and 0 mm^2^. These value for the aforementioned coating in the presence of Escherichia coli bacteria was about 31 mm and 48 mm^2^, respectively. Also, HA-based composite coatings show higher antibacterial properties in the presence of Escherichia coli bacteria compared to those in the presence of Staphylococcus aureus. This difference in diameter and surface area of the inhibition zone for two types of bacteria is related to silver nanoparticles. In fact, the difference between the response of gram-positive and gram-negative bacteria in silver nanoparticles is related to the difference in the structure of their cell wall. Gram-negative bacteria have thinner cell walls, and they have a layer of lipopolysaccharide on their outer surfaces. This layer contains negative charges and facilitates the interaction between silver nanoparticles (positive charge) and these bacterial cells. The attachment of nanoparticles to the cell surfaces initially perforates the wall. Then as the nanoparticles enter the bacterial cell, it eventually leads to the death of the bacteria [55]. By considering these results, it can be said that applying of HA-Ag composite coatings can improve the cell viability and cell attachment, bioactivity, and antibacterial properties of the samples.

### Corrosion behavior

#### OCP Study

**Fig. 13:**
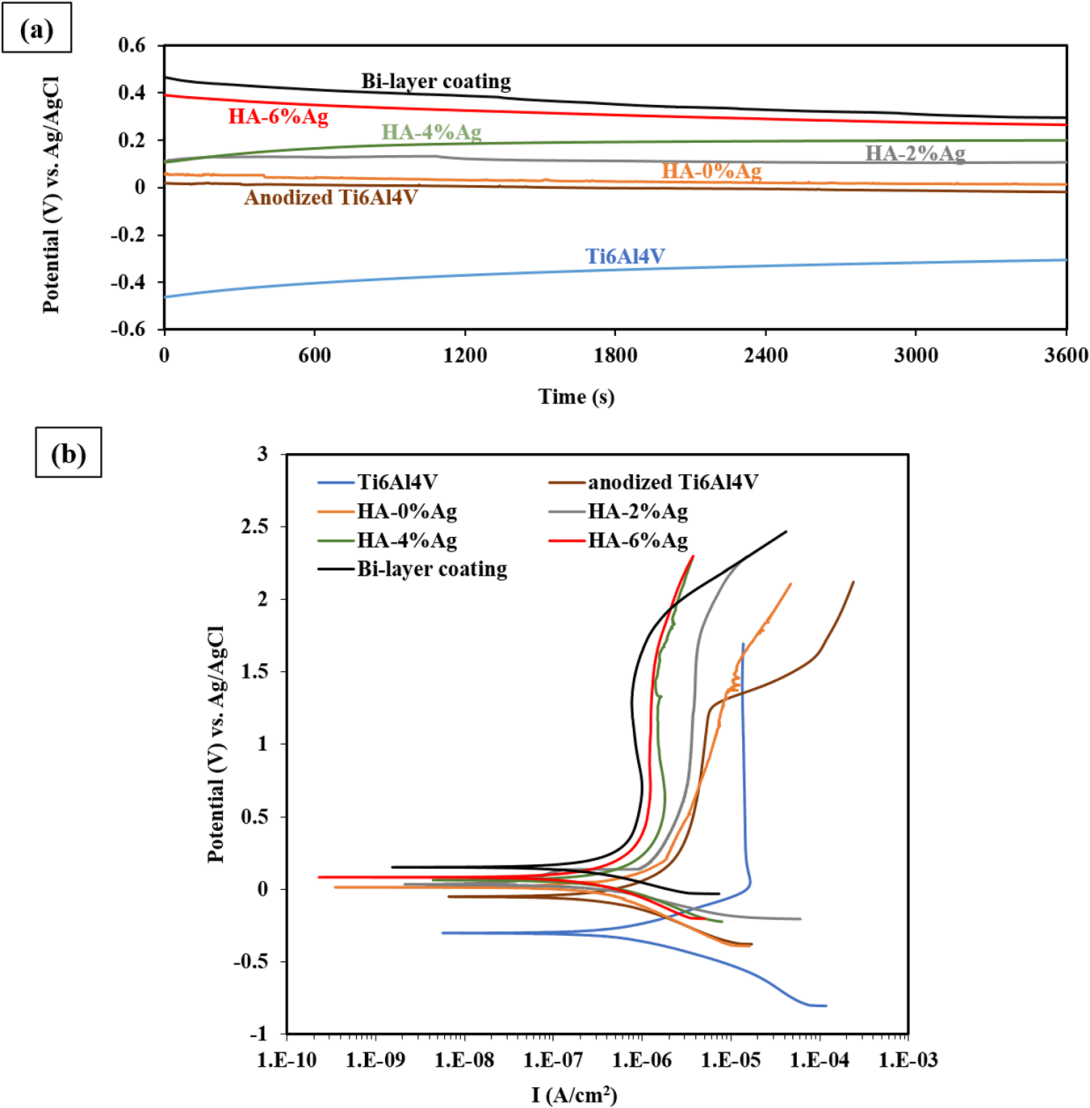
**a** Variation of OCP with time and (**b**) The graphs of tafel polarizations of the samples in PBS at 25 °C

Figure 13a, b, respectively, illustrate the variation of open circuit potential (OCP) with time and the Tafel polarization graphs of the samples in PBS at 25 °C. The extracted data from Fig. 13 are also reported in Table 4. The OCP in all samples reaches to constant values after one h immersion in PBS solution. This figure shows that the lowest OCP is for Ti6Al4V alloy. The anodizing process and applying the HA-Ag coatings on Ti6Al4V increases the OCP of specimens. Higher values of OCP for the aforementioned samples indicate their higher thermodynamic stability. In other words, the corrosion tendency reduces after anodizing and also coating. The oxide layer created by anodizing, as well as the HA-based composite coatings behave like ceramics and because of their chemical neutrality, the coated specimens show lower corrosion tendencies as compared with that of the Ti6Al4V [56].

**Table 4:** Corrosion characteristics (obtained from OCP and tafel polarization curves) of samples

sample	OCP (V_SCE_)	Ecorr (V_SCE_)	Icorr (μA.cm^−2^)	βa (V.dec^−1^)	β_c_ (V.dec^−1^)	R_p_ (Ω.cm^2^)
Ti6Al4V	−0.3	−0.33	0.9	0.13	0.20	38012.20
Anodized Ti6Al4V	−0.01	−0.05	0.33	0.17	0.21	123616.58
HA-0%Ag	0.01	0.02	0.29	0.17	0.22	143586.98
HA-2%Ag	0.1	0.05	0.21	0.16	0.13	148303.74
HA-4%Ag	0.19	0.09	0.18	0.12	0.13	150528.30
HA-6%Ag	0.26	0.15	0.14	0.10	0.13	175304.69
Bi-layer coating	0.29	0.2	0.12	0.13	0.15	252000.50

Figure 13a shows that the OCP value of coated samples are higher than that of the anodized layer. This is due to the porous nature (Fig. 2a) and fairly low thickness of the anodized layer (Fig. 2b) compared to HA-based composite coatings (Fig. 5a). Low thickness and porosities in the anodized layer make it easier for PBS solution to diffuse to Ti6Al4V substrate during the corrosion test. Consequently, by interaction of Ti6Al4V with PBS solution, the OCP value of this layer is reduced. In addition, the shift of OCP to higher values by increasing of the silver content in the coatings is noticed in Fig. 13a. This can probably be attributed to the lower corrosion tendency of silver particles as a noble component [57].

Compared to the anodized and HA-Ag coatings, the bi-layer coating has a higher OCP, which indicates a lower corrosion tendency. Since this bi-layer coating (HA-4%Ag on the anodized layer) is thicker (approximately 20 µm) than the other coatings, its thermodynamic stability must be higher than other samples. The values of the OCP of samples are reported in Table 4.

#### Tafel polarization curves investigation

The potentiodynamic polarization of samples that were conducted in PBS solution at 25 °C are shown in Fig. 13b. The corrosion potential (Ecorr), corrosion current density (Icorr), anodic beta coefficient (βa), and cathodic beta coefficient (βc) were determined by nova software and reported in Table 4.

According to Fig. 13b and Table 4, the Ti6Al4V alloy has the maximum corrosion current density (Icorr), and after anodizing process and coating of the samples, it reduces. The low Icorr in anodized and coated samples compared to Ti6Al4V are due to the formation of ceramic natured layers on these specimens with non-conductive behavior.

Also, Fig. 13b and Table 4 display that oxide and bio-ceramic layers formed by anodizing and EPD process cause the shifting of corrosion potential to more noble values. This is due to the low corrosion tendency of these layers. H. Farnoush et al. [58] illustrated in their research that the EPD of HA-TiO2 composite coating on Ti6Al4V improves the corrosion resistance of the material. In other words, this coating effectively reduces the corrosion current density and increases corrosion potential.

The polarization curves show that the anodized layer is less effective than the composite coatings in reducing corrosion current density. Also, it should be noted that the increase of silver nanoparticles concentration in the coating suspension has little effect on decreasing corrosion current density.

Corrosion potential is a thermodynamic parameter, while corrosion current density is considered as a kinetic parameter. According to Eq. 5, the corrosion resistance of the materials is mainly determined by corrosion current density [59–62].

5 $${\rm{Rp}}=(\beta {\rm{a}}.\beta {\rm{c}})/(2.303\times {\rm{Icorr}}\times (\beta {\rm{a}}+\beta {\rm{c}}))$$

In Eq. 5, Rp is the polarization resistance, Icorr is the corrosion current density, and βa and βc are the anodic and cathodic beta coefficients, respectively. The values of the polarization resistance of the samples were calculated using Eq. 5 are reported in Table 4. This Table represents that after anodizing and EPD processes, the corrosion resistance of the samples has increased significantly. According to polarization curves, increasing of silver nanoparticles in composite coating decreases the corrosion current density and finally, the polarization resistance of the specimen increases. This is believed to be due to the blocking of the pores by highly noble clusters [57].

As mentioned in Section 3.5.2, the release of the elements after corrosion reactions affects the spreading and adhesion of osteoblast cells to the surface. The corrosion test results of the samples shown in Fig. 13b and Table 4 confirm the cell adhesion to the surface (Fig. 9). In other words, the higher spreading of cells on the anodized and coated surfaces is due to the high corrosion resistance of these layers. So, HA-based composite coatings as a bio-ceramic layer can increase the corrosion resistance of the samples by reducing corrosion current density (Icorr). These layers also reduce the corrosion tendency of the specimens.

Overall, despite the improvement of biological and electrochemical properties, the (HA-Ag) composite coatings created by the EPD method have poor adhesion to the substrate. This challenge can reduce the tribological properties of coatings and limit their use in the body environment. Therefore, some approaches such as suitable sintering of coatings or creating an intermediate layer should be adopted to increase the adhesion of these coatings to the substrate.

## Conclusions

In this study, HA-Ag composite coatings were fabricated by the EPD method on bare and anodized Ti6Al4V. The coatings were applied on the substrate to improve the biological and electrochemical behaviors. The results can be summarized as follows:

1. HA-Ag composite coatings significantly improved the biological properties of Ti6Al4V alloy.
2. HA-4%Ag composite coating and bi-layer coating (HA-4%Ag on anodized Ti6Al4V) had the highest cell viabilities, and HA-6%Ag showed the highest antibacterial properties.
3. Cell adhesion and bioactivity of HA-Ag coatings were almost the same. Moreover, by increasing the silver content in the coating suspensions, no significant difference was observed in the mentioned cases.
4. After anodizing process, cell viability, cell adhesion, and corrosion resistance increased, but this layer had no effect on increasing bioactivity and antibacterial properties.
5. The corrosion resistance of Ti6Al4V specimen increased remarkably after applying HA-Ag coatings and the bi-layer coating (HA-4%Ag on anodized Ti6Al4V) had a higher corrosion resistance than those of the others.
